# *Cyperus prophyllatus*: An endangered aquatic new species of *Cyperus* L. (Cyperaceae) with a exceptional spikelet disarticulation pattern among about 950 species, including molecular phylogenetic, anatomical and (micro)morphological data

**DOI:** 10.1371/journal.pone.0249737

**Published:** 2021-06-09

**Authors:** André Rodolfo de Oliveira Ribeiro, Luciana Pereira-Silva, Jéssika Paula Silva Vieira, Isabel Larridon, Vinicius Santos Ribeiro, Guilherme Felitto, Geovane Souza Siqueira, Anderson Alves-Araújo, Marccus Alves

**Affiliations:** 1 Departamento de Fitotecnia, Universidade Federal do Ceará, Campus do Pici, Fortaleza, Ceará, Brazil; 2 Programa de Pós-Graduação em Biologia de Fungos, Algas e Plantas, Departamento de Botânica, Universidade Federal de Santa Catarina, Florianópolis, Santa Catarina, Brazil; 3 Royal Botanic Gardens, Kew, Surrey, United Kingdom; 4 Departamento de Botânica, Universidade de Brasília, Campus Universitário Darcy Ribeiro, Brasília, Distrito Federal, Brazil; 5 Systematic and Evolutionary Botany Lab, Department of Biology, Ghent University, Gent, Belgium; 6 Faculdade de Educação, Universidade Federal do Ceará, Fortaleza, Ceará, Brazil; 7 Reserva Natural Vale, Linhares, Espírito Santo, Brazil; 8 Departamento de Ciências Agrárias e Biológicas, Universidade Federal do Espírito Santo, São Mateus, Espírito Santo, Brazil; 9 Departamento de Botânica, Universidade Federal de Pernambuco, Recife, Pernambuco, Brazil; Universidad Pablo de Olavide, SPAIN

## Abstract

*Cyperus prophyllatus*, an endangered new species of *Cyperus* (Cyperaceae) from an aquatic ecosystem of the Atlantic Forest, Espírito Santo State, southeastern Brazil, is described and illustrated. The spikelet morphology of *Cyperus prophyllatus* is unique among the c. 950 species of *Cyperus* in having both a conspicuous spikelet prophyll and a corky rachilla articulation, which remain persistent at the base of the spikelet after disarticulation. Our molecular phylogenetic data support the placement of *C*. *prophyllatus* in the C_3_ *Cyperus* Grade and more precisely in the clade representing *Cyperus* sect. *Oxycaryum*, which also includes *C*. *blepharoleptos* and *C*. *gardneri*. Anatomical and (micro)morphological analyses corroborate the phylogenetic results, provide a better understanding of ecology and taxonomy, as well as reveal compatibility of structures with survival and dispersion in aquatic environments. A distribution map, table with distinctive characters of allied species, and conservation status are made available.

## Introduction

*Cyperus* L. is the second largest genus in Cyperaceae and the most diverse in tribe Cypereae, comprising about 950 species [1–3]. *Cyperus* has a worldwide distribution, with about 130 species registered in Brazil, where they occur in diverse habitats and vegetation types [4,5]. In aquatic ecosystems, *Cyperus* species contribute to water purification, reducing microbiological contaminants, improving physical and chemical parameters, as well as help to control erosion and sedimentation into the waterbodies [6,7].

Recent studies of *Cyperus* based on morphology, anatomy, physiology, and molecular phylogenetic data subdivided the genus into two groups, C_3_ *Cyperus* Grade and C_4_ *Cyperus* Clade [3,8–13]. The C_3_ species of *Cyperus* compose a grade of generally well-circumscribed *Cyperus* sections with eucyperoid anatomy using the C_3_ photosynthetic pathway, whilst the C_4_ species of *Cyperus* have been consistently retrieved in a well-supported clade with chlorocyperoid anatomy using C_4_ photosynthetic pathway [3,9–14].

The genus *Cyperus* is recognized by leaves in spiral phyllotaxis, inflorescence terminal, glumes distichously or rarely spirally arranged, flowers usually hermaphrodite, without perianth, style bifid or trifid, and achenes lenticular or trigonous [3,9–18]. Notwithstanding that the *Cyperus* species share morphological characters that allow the distinction from other genera, there is high morphological variability among subgenera, sections, and species, as well as important taxonomic value in the leaf blade and culm shape, inflorescence type, spikelet disarticulation pattern, glume (shape and color), style branching, stamens (number and shape), and achene (color, shape and surface) [3,10,13,15,16,19–24].

The spikelet disarticulation pattern refers to the diaspore, its morphology, constitutive organs, and the mode in which it disarticulates from the plant [15,16,19,21]. This has a direct relationship with the dispersal of reproductive structure and represents an adaptation to its native environmental conditions [15,16,19,21]. Within the spectrum of variation of the spikelet disarticulation pattern in *Cyperus*, there is preponderance of species with persistent glumes, deciduous spikelets, and rachilla articulate above the spikelet prophyll or species with glumes gradually deciduous from the base to the apex of the spikelet with the rachilla disarticulating belatedly after the fall of the glumes [3,13,10,15,16,19,21,25,26].

In the New World, Brazil is among the areas of high diversity and endemism in *Cyperus* with several new species recently published, mainly from aquatic environments [26–33]. In Brazil, the aquatic ecosystems suffer severe degradation because of activities such as illegal logging, mining, ranching, and agriculture [34–37], which reduce vegetation cover, decrease rainfall, increase evaporation and incidence of drought, and thus trigger decline in populations or even extinction of species not yet discovered [36–40].

Seeking to expand knowledge about the diversity and evolution of the genus *Cyperus* and to supply information for continuity in conservation of natural ecosystems in Brazil, the purpose of the present study was to describe a new species of *Cyperus* from Southeastern Brazil, and providing molecular phylogenetic, anatomical, (micro)morphological and ecological data for this species.

## Material and methods

### Taxonomy and morphological analysis

The first specimens of the new species were found in the herbaria CVRD and UFP (abbreviations according to Thiers [41]), but it was not possible to describe neither the plant habit nor the rhizome type. Based on data from these first collection, we conducted two field expeditions to Espírito Santo State in September 2018 (no flowering plants were found) and September 2019 (with flowers and fruits available), and therefore it was possible to observe and study the rhizome and plant habit of the new species in its natural habitat. The field expeditions and site access were authorized by Marcio Elias Santos Ferreira, manager of Reserva Natural Vale.

In addition, we analyzed the specimen collections deposited in the herbaria of Espírito Santo State (CVRD, MBML,VIES, SAMES) and other states of Brazil (ASE, CEN, EAC, FLOR, HUEFS, IBGE ICN, MAC, MOSS, RB, SP, UB, UFP, and UFRN), besides images of exsiccatae from the Brazilian herbaria available in SpeciesLink [42]. High resolution images of the type specimens of *Cyperus* species present in several international herbaria (B, C, G, K, P, MO, NY, US) were also examined. The morphological descriptions of the character states followed Radford *et al*. [43].

Conservation status was assessed based on IUCN Red List criteria [44] with area of occupancy (AOO) and extent of occurrence (EOO) estimated using the Geospatial Conservation Assessment Tool [45]. Distribution map of new species and allied species was made using software QGIS v.3.16.0 (https://qgis.org).

### Nomenclature

The electronic version of this article in Portable Document Format (PDF) in a work with an ISSN or ISBN will represent a published work according to the International Code of Nomenclature for algae, fungi, and plants, and hence the new names contained in the electronic publication of a PLOS article are effectively published under that Code from the electronic edition alone, so there is no longer any need to provide printed copies.

In addition, new names contained in this work have been submitted to IPNI, from where they will be made available to the Global Names Index. The IPNI LSIDs can be resolved and the associated information viewed through any standard web browser by appending the LSID contained in this publication to the prefix http://ipni.org/. The online version of this work is archived and available from the following digital repositories: PubMed Central, LOCKSS.

### Molecular data and phylogenetic analysis

Taxon sampling was based on recent classification of *Cyperus* [3,10–14], including C_3_ and C_4_ species. DNA sequence data of ETS, ITS, *rpl32-trnL*, and *trnH-psbA* markers published in previous studies [1,3,10,11,13,46] are used in this study. In addition, we obtained new DNA sequences of the new species and of the *C*. *appendiculatus* (Brongn.) Kunth for the ITS marker. The final dataset comprises 35 accessions, representing 31 species of *Cyperus* and four outgroup species (*Ficinia gracilis* Schrad., *Isolepis fluitans* (L.) R.Br., *Scirpoides holoschoenus* (L.) Soják, *and S*. *mexicana* (C.B.Clarke ex Britton) Goetgh. ex C.S.Reid & J.R.Carter). The species names, voucher information, and GenBank accession numbers are listed in Table 1.

**Table 1:** List of the samples, voucher, and GenBank accessions numbers for the species used in the phylogenetic analysis.

Species	Voucher	ITS	ETS	*trnH-psbA*	*rpl32-trnL*	Reference
*Cyperus andinus* Palla ex Kük.	Gonzalez 8114 (LSU)	KX306830		KX405725	KX405621	[1]
*Cyperus appendiculatus* (Brongn.) Kunth	Figueira 848 (UB)	MW520742				This study
*Cyperus blepharoleptos* Steud.	Reid 7796 (LSU)	KF150596		KX405834	KX405720	[1]
*Cyperus blepharoleptos* Steud.	Zardini 18398 (GENT)		HQ705942		HQ705875	[13]
*Cyperus brasiliensis* (Kunth) Bauters	Larridon *et al*. 2010–0304 (GENT)		HE993954	HE993894	HE993685	[13]
*Cyperus compressus* L.	Reid and Carter 7761 (LSU)	KF193575		KX405735	KX405628	[1]
*Cyperus croceus* Vahl	Reid 7501 (LSU)	KF150543		KX405737	KX405631	[1]
*Cyperus cuspidatus* Kunth	Reid and Carter 7760 (LSU)	KF150544		KX405739	KX405632	[1]
*Cyperus debilissimus* Baker	Larridon *et al*. 2010–0103 (GENT)		HQ705933	HQ705808	HQ705866	[11]
*Cyperus difformis* L.	Gonzalez 8127 (LSU)	KX306836		KX405741	KX405634	[1]
*Cyperus erinaceus (*Ridl.) Kük.	Faden *et al*. 96/358 (K)		HQ705969	HQ705836	HQ705899	[13]
*Cyperus esculentus* L.	Reid 7481 (LSU)	KF150553		KX405754	KX405648	[1]
*Cyperus flavescens* L.	Reid 7576 (LSU)	KF150598		KX405759	KX405655	[1]
*Cyperus fuscus* L.	Reid 7788(LSU)	KF150555		KX405761	KX405657	[1]
*Cyperus gardneri* Nees	Schessl 3316 (GENT)		HQ705943		HQ705876	[11]
*Cyperus haspan* L.	Muasya & Muthama 1269 (EA)		HQ705927	HQ705803	HQ705860	[11]
*Cyperus hyalinus* Vahl	Muasya 2490 (EA)		HQ705967	HQ705834	HQ705897	[11]
*Cyperus iria* L.	Gonzalez 8131 (LSU)	KX306848		KX405772	KX405666	[1]
*Cyperus ligularis* L.	Gonzalez 8139 (LSU)	KX306850		KX405779	KX405672	[1]
*Cyperus luzulae* (L.) Retz.	Reid 7808 (LSU)	KF150565		KX405781	KX405674	[1]
*Cyperus pectinatus* Vahl	Larridon *et al*. 2010–0265 (GENT)		HQ705936	HQ705810	HQ705869	[11]
*Cyperus pedunculatus* (R.Br.) J.Kern	Faden *et al*. 96/48 (K)		HQ705968	HQ705835	HQ705898	[11]
*Cyperus prophyllatus* A.R.O.Ribeiro, Pereira-Silva & M.Alves	Ribeiro *et al*. 487 (CVRD)	MW520743	Submitted to GenBank			This study
*Cyperus prophyllatus* A.R.O.Ribeiro, Pereira-Silva & M.Alves	Ribeiro *et al*. 490 (CVRD)	MW520744	Submitted to GenBank			This study
*Cyperus pseudokyllingioides* Kük.	Larridon *et al*. 2010–0261 (GENT)		HQ705941	HQ705814	HQ705873	[11]
*Cyperus reduncus* Hochst. ex Boeckeler	Malaisse & Goetghebeur 1171 (GENT)		HQ705938	HQ705811	HQ705871	[11]
*Cyperus rotundus* L.	Abbott 23635 (FLAS)	KF150582		KX405810	KX405700	[1]
*Cyperus sesquiflorus* (Torr.) Mattf. & Kük.	Reid and Carter 7753 (LSU)	KF150591		KX405829	KX405714	[1]
*Cyperus subsquarrosus* (Muhl.) Bauters	Reid 7577 (LSU)	KF150595		KX405833	KX405719	[1]
*Cyperus surinamensis* Rottb.	Reid 7478A (LSU)	KF150585		KX405818	KX405707	[1]
*Cyperus textilis* Thunb.	Goetghebeur 11517 (GENT)		HQ705951	HQ705820	HQ705881	[11]
*Ficinia gracilis* Schrad.	Muasya 2713 (BOL)		HQ705902	HQ705784		[11]
*Isolepis fluitans* (L.) R.Br.	Muasya & Knox 3195 (EA)		HQ705901	HQ705783		[11]
*Scirpoides holoschoenus* (L.) Soják	Goetghebeur 11520 (GENT)		HQ705900	HQ705782	HQ705837	[11]
*Scirpoides mexicana *(C.B.Clarke ex Britton) Goetgh. ex C.S.Reid & J.R.Carte	Gonzalez 8112 (LSU)			KX405827	KX405712	[1]

Total DNA was extracted from 15–20 mg of silica-dried leaf tissue using a modified CTAB (cetyltrimethylammonium bromide) protocol [47]. PCR conditions for amplification and primers followed Reid et al. [46]. Cycle sequencing was performed with the same primers used for amplification and Sanger sequencing was conducted at Jodrell Laboratory of the Royal Botanic Gardens Kew (London, UK). DNA sequences were assembled in Geneious v.7.1.9 [48] per marker and aligned using the MAFFT v.7 [49], with subsequent manual adjustment in PhyDE v.0.9971 [50].

Phylogenetic hypotheses were reconstructed using both Bayesian Inference (BI) and Maximum Likelihood (ML) approaches. We first inferred gene trees for each of the four regions, which were concatenated afterwards since no conflict was found for supported nodes. The best models of nucleotide substitution were determined with PartitionFinder2 [51], using the Akaike Information Criterion (AIC), in this case, each marker was treated as a separate partition. The GTR+G model was determined to be the best model for all partitions. Bayesian Inference was performed using MrBayes v.3.2.7 [52]. Four independent runs of four Monte Carlo Markov chains each were conducted with 20,000,000 generations, sampling every 1000 generations, and 25% burn-in. We assessed convergence and effective sample size (ESS) values using Tracer v.1.7 [53]. ML analyses were performed with RAxML v.8.2.12 [54] using the rapid bootstrap with 1000 replicates, combined with a search of the best-scoring ML tree, and the remaining options set to default. All analysis of BI, ML and PartitionFinder2 were executed in the CIPRES Science Gateway [55]. Outputs of BI and ML were read using Figtree v.1.4.4 (http://tree.bio.ed.ac.uk/software/figtree/).

### Micromorphology and Scanning Electron Microscopy (SEM)

The samples were prepared from leaf blade, ligule, spikelet prophyll, glumes, and achenes sourced from existing herbarium specimens and specimens newly collected by us. Deformed and flaccid immature achenes and spikelets were not considered. The samples were mounted onto aluminum metal stubs using carbon double-stick tape and sputter-coated with platinum without pre-treatment. The images were captured using a SEM (Jeol JSM 7001S) under 15 KV at the Electronic Microscope Laboratory of the University of Brasília. The descriptive terminology followed Ellis [56], Haines & Lye [19], Hefler & Longhi-Wagner [57], and Shalabi & Gazer [58].

### Anatomy

The leaf samples were obtained from the middle third of the leaf blades collected at field or from the herbarium specimens. At least three leaf blades were analyzed in each accession. The samples were stored in the ethanol aqueous solution 70% (v / v) at 4°C and after rehydrated in the glycerol aqueous solution 1: 1 (v / v), until the preparation of free-hand sections [59].

The leaf transverse sections were cut in the table microtome (type R. Jung A. G. Heidelberg) available in Laboratório de Anatomia Vegetal in Universidade de Brasília (UnB). The best sections were selected and bleached with sodium hypochlorite aqueous solution 20% (m / v), and after 50% (m / v) for at least five minutes or until the complete discoloration of the tissues [60].

Before the staining, the samples were dehydrated in progressive ethanol concentrations in aqueous solution (50%, 70%, 92,6%, and 100% [v/v]) for fixation of the stain in the butyl acetate. The double staining was performed with safranine aqueous solution 1% (m / v) and alcian blue aqueous solution 1% (m / v). The permanent slides were mounted with colorless glass varnish, according the protocol described by Paiva et al. [61]. The descriptive terminology followed Ellis [62].

The images were acquired with the Leica DM 750 microscope in Laboratório de Criptógamas in Universidade de Brasília. The examination of the images and the evaluation of the tissues and cells were executed with the software Leica Application Suite (version 4.5).

## Results

### Taxonomic treatment

**Fig 1:**
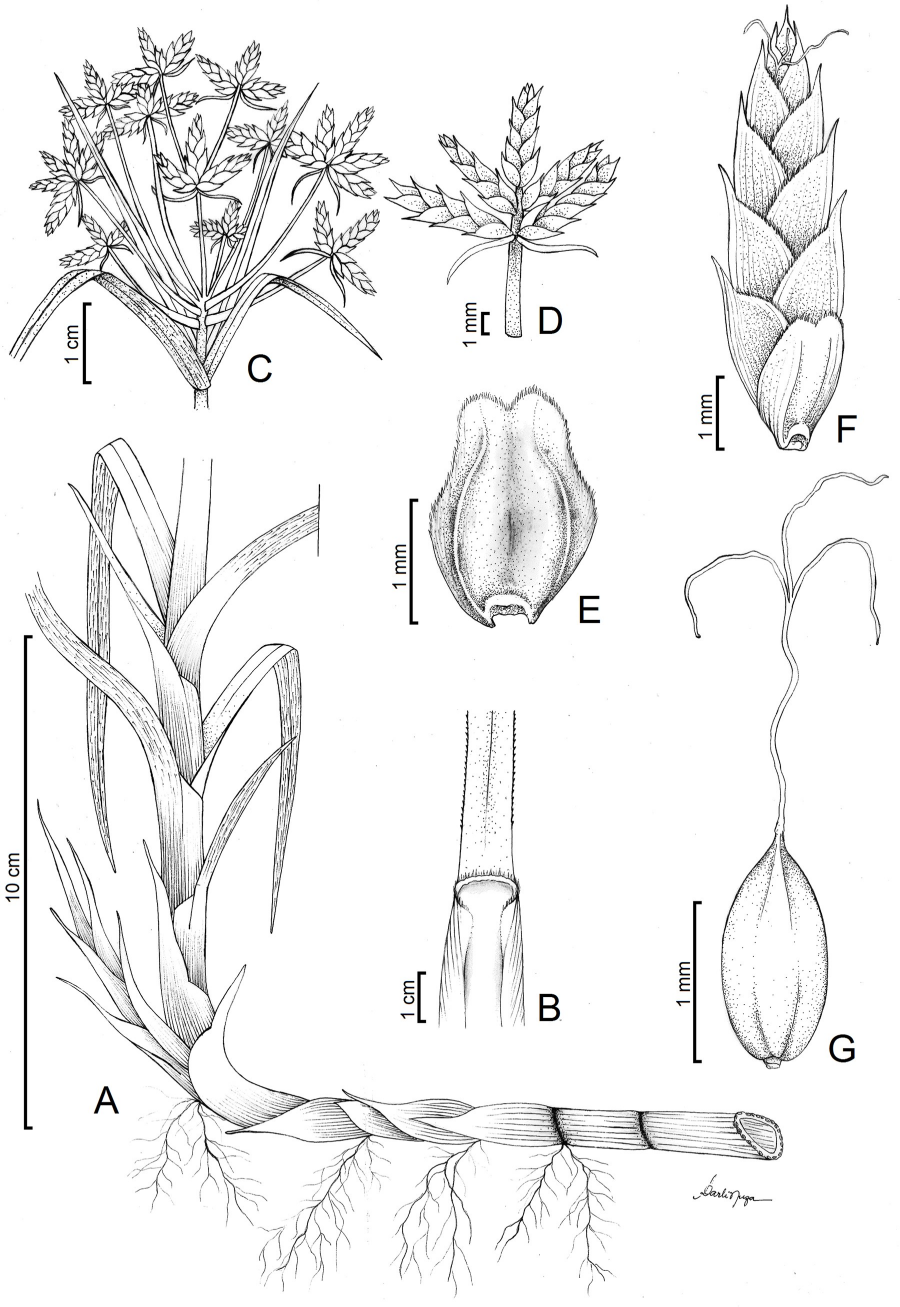
Botanical illustration of *Cyperus prophyllatus*, *sp*. *nov*. Drawn by Darli Nuza from holotype *A*.*R*.*O*. *Ribeiro et al*. *487* (CVRD). (A) Plant habit showing floating rhizome leptomorph. (B) Detail of leaf exposing the leaf sheath below, ligule at middle, and leaf blade above. (C) Inflorescence. (D) Detail of spike on ultimate order of the inflorescence. (E) Spikelet prophyll with a corky rachilla articulation persistent at base. (F) Spikelet. (G) Achene with trifid style persistent on apex.

**Fig 2:**
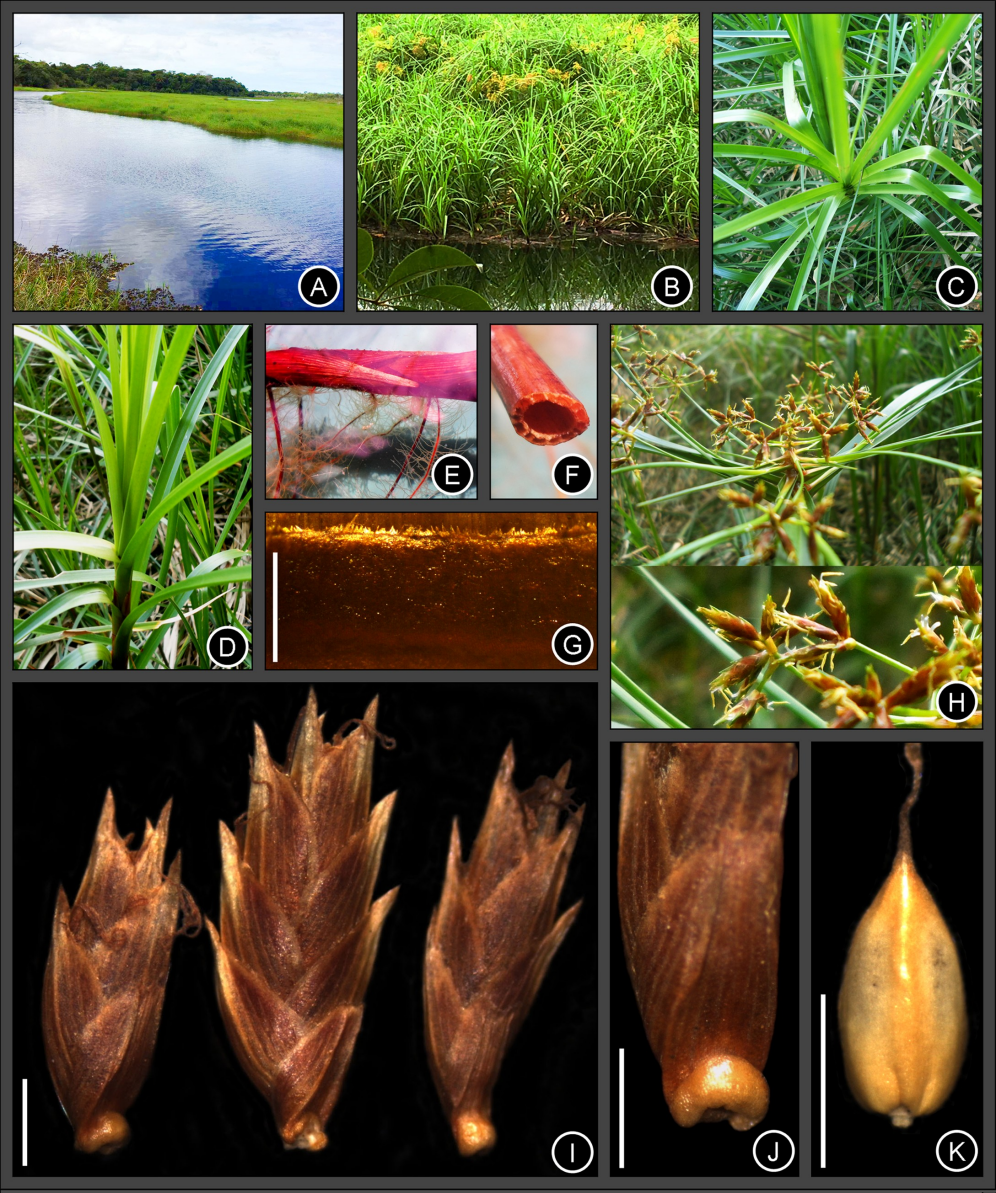
Photographs of *Cyperus prophyllatus*, *sp. nov.* Taken from holotype *A.R.O. Ribeiro et al. 487* (CVRD). (A–B) Floating population in its natural habitat. (C–D) Leaves in spiral phyllotaxis spacing along the culm. (E–F) Leptomorph floating rhizome. (E) Detail showing cataphylls and roots. (F) Cross section revealing hollow internode. (G) Ligule with ciliate hairs at apex. (H) Inflorescence with detail of ultimate order branch below. (I) Spikelets showing its prophyll attached at base. (J) Detail of protuberant rachilla articulation persistent at the base of the spikelet prophyll. (K) Achene with style persistent at apex. Scale bars: G, I–K = 1 mm.

**Fig 3:**
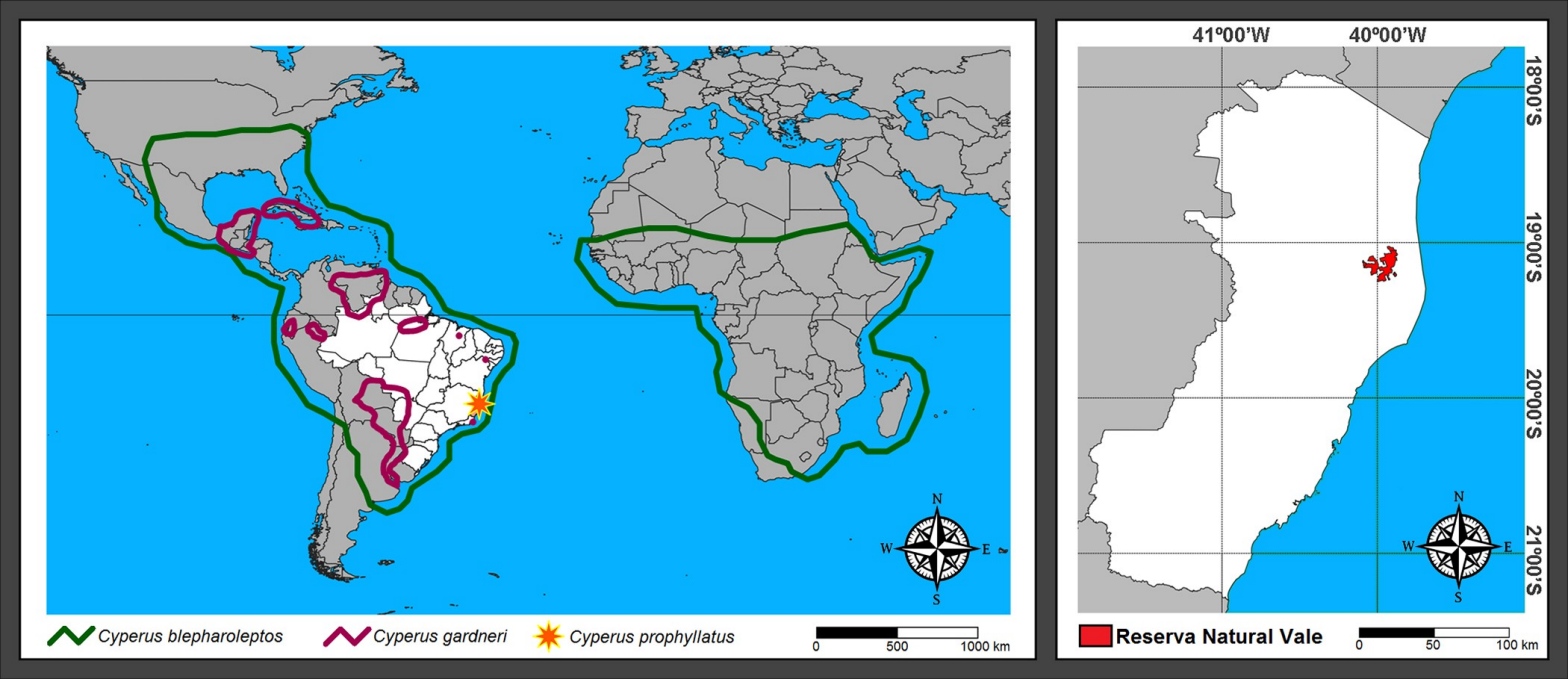
Distribution map of *Cyperus prophyllatus*, *sp*. *nov*., and allied species.

**Fig 4:**
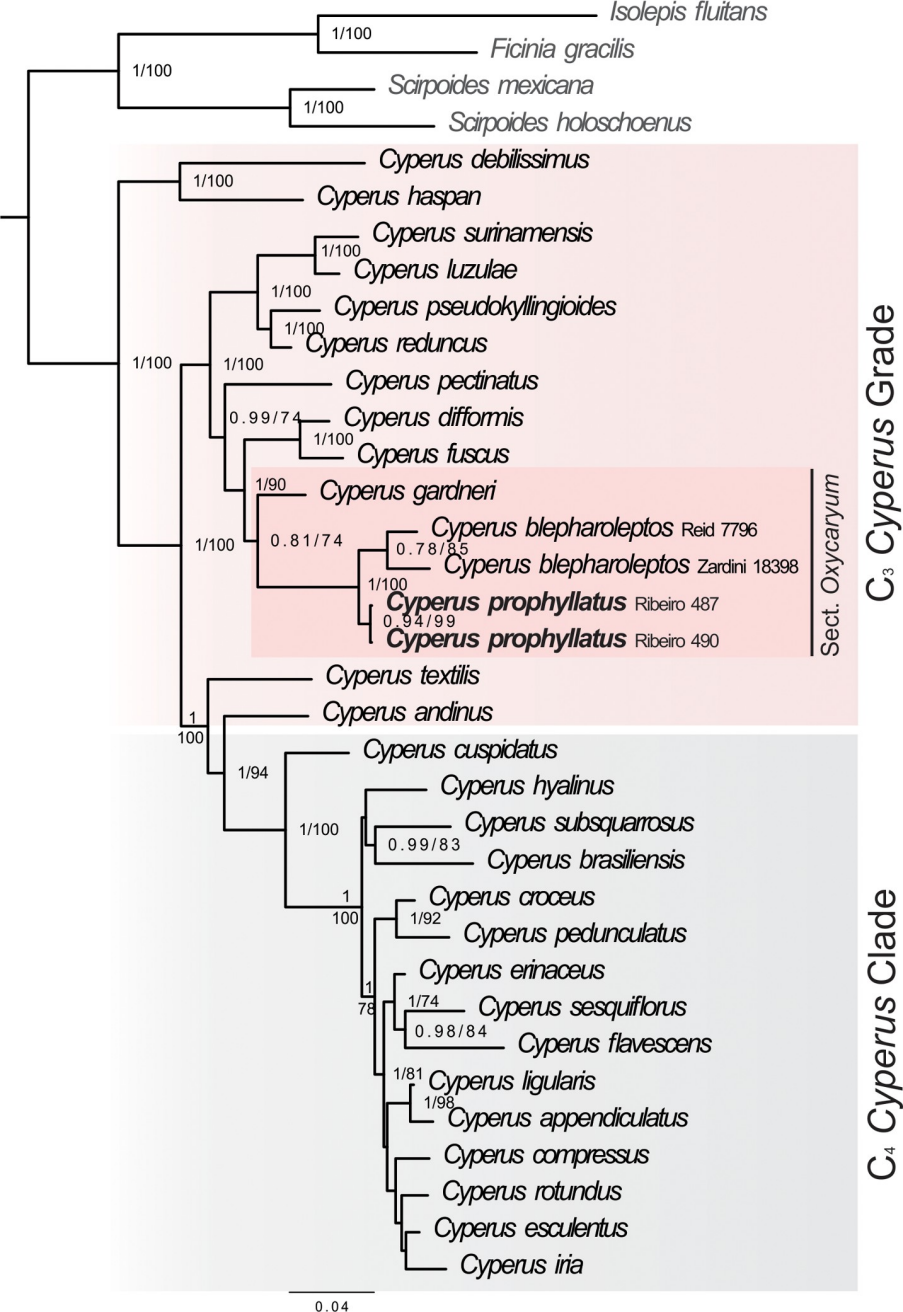
50% majority consensus BI tree, including *Cyperus prophyllatus* and allied species. This BI tree is resulting from the combined dataset analysis with the associated posterior probability (PP) values and the bootstrap values of the ML tree. Posterior probabilities greater than 0.75 and bootstrap values greater than 70% are shown. The newly described *Cyperus prophyllatus* is indicated in bold.

**Fig 5:**
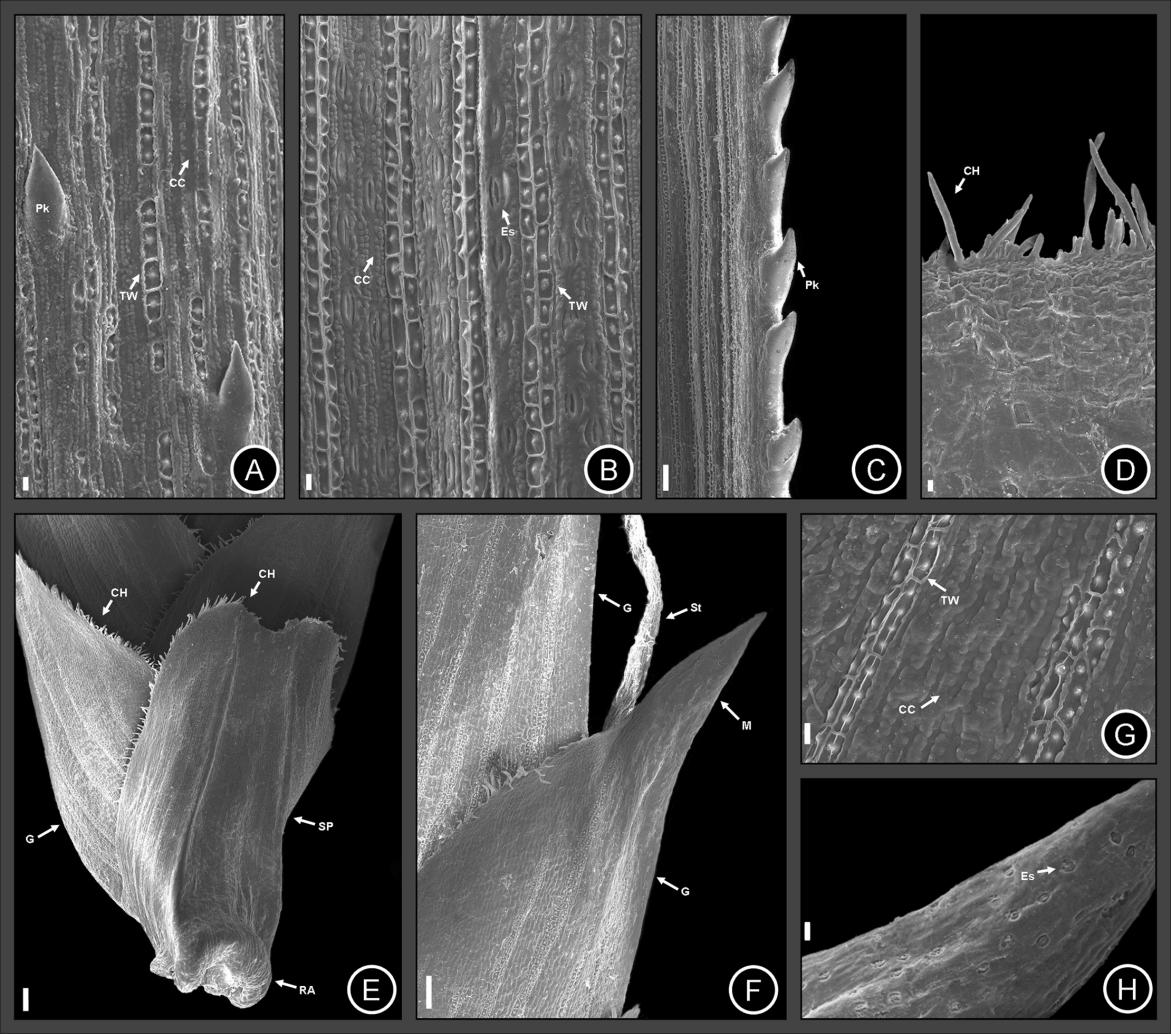
Micrographs taken by Scanning Electron Microscopy (SEM) from leaves and spikelets of *Cyperus prophyllatus*, *sp*. *nov*. Taken from holotype *A*.*R*.*O*. *Ribeiro et al*. *487* (CVRD). (A–C) Leaf blade. (A) Adaxial surface. (B) Abaxial surface. (C) Leaf blade margin surface viewed from the abaxial side. (D) Ligule surface viewed from the adaxial side. (E) Spikelet prophyll surface viewed from the abaxial side showing a protuberant articulation at base, ciliate single-celled hairs at the margins, and some glumes above the prophyll apex. (F–H) Glume surface viewed from the abaxial side. (F) View of glume showing ciliate single-celled hairs at the margins, the glabrous mucron at the apex, and the style arising from the glume axil. (G) Detail of glume epidermis showing rows of thick-walled epidermal cells tabular or square-shaped and the common epidermal cells. (H) Detail of glume mucron showing stomata. CC = common epidermal cell, CH = ciliate single-celled hair, Es = Stomate, G = Glume, M = mucron, Pk = prickle, RA = rachilla articulation, SP = spikelet prophyll, St = Style, TW = thick-walled epidermal cell. Scale bars: A, B, D, G = 10 μm. C = 50 μm. E–F = 100 μm. H = 20 μm.

**Fig 6:**
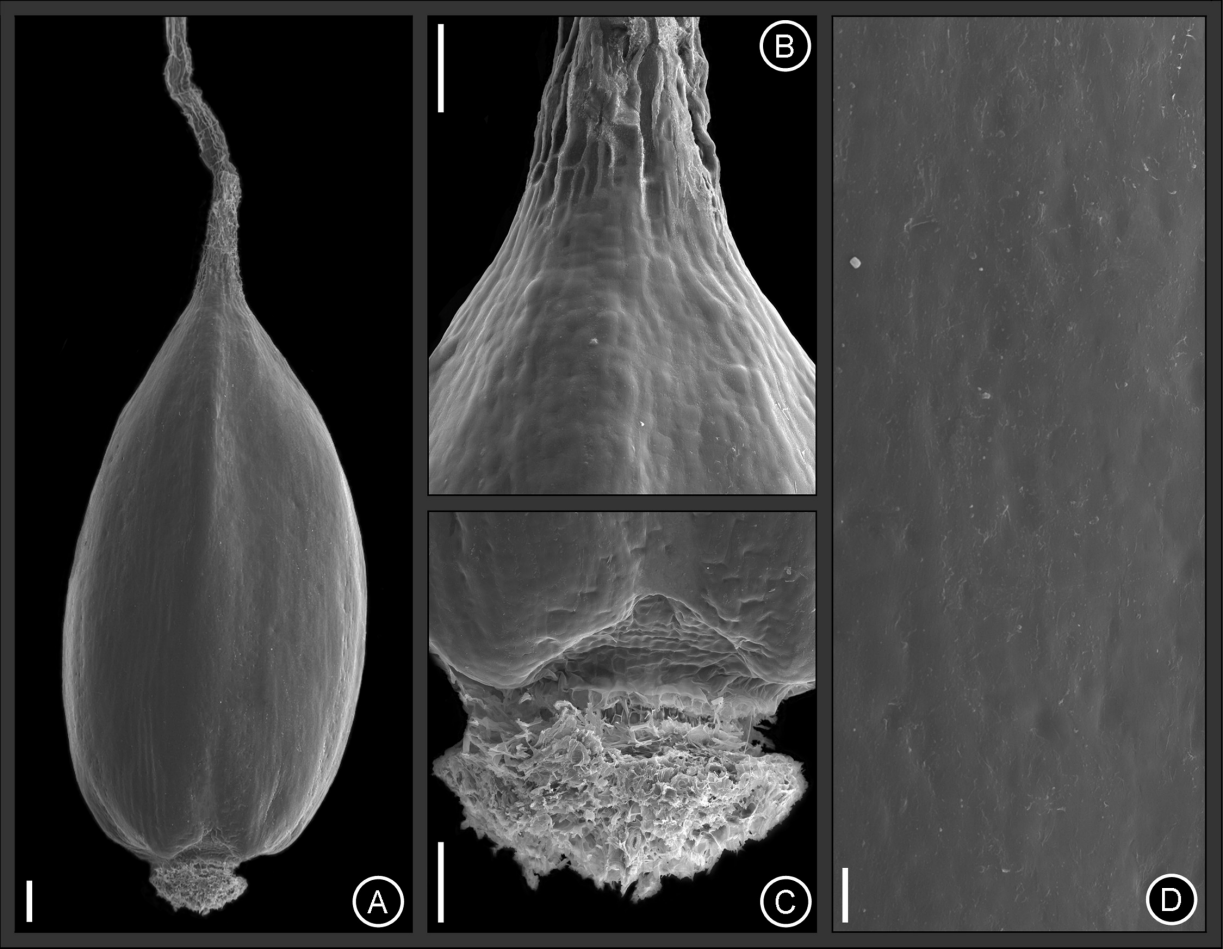
Micrographs taken by Scanning Electron Microscopy (SEM) from the achene surface of *Cyperus prophyllatus*, *sp*. *nov*. Taken from holotype *A*.*R*.*O*. *Ribeiro et al*. *487* (CVRD). (A) Entire view showing style persistent at apex. (B) Achene apex. (C) Achene base. (D) Detail of the Achene surface. Scale bars: A. 100 μm. B–C. 50 μm. D. 10 μm.

**Fig 7:**
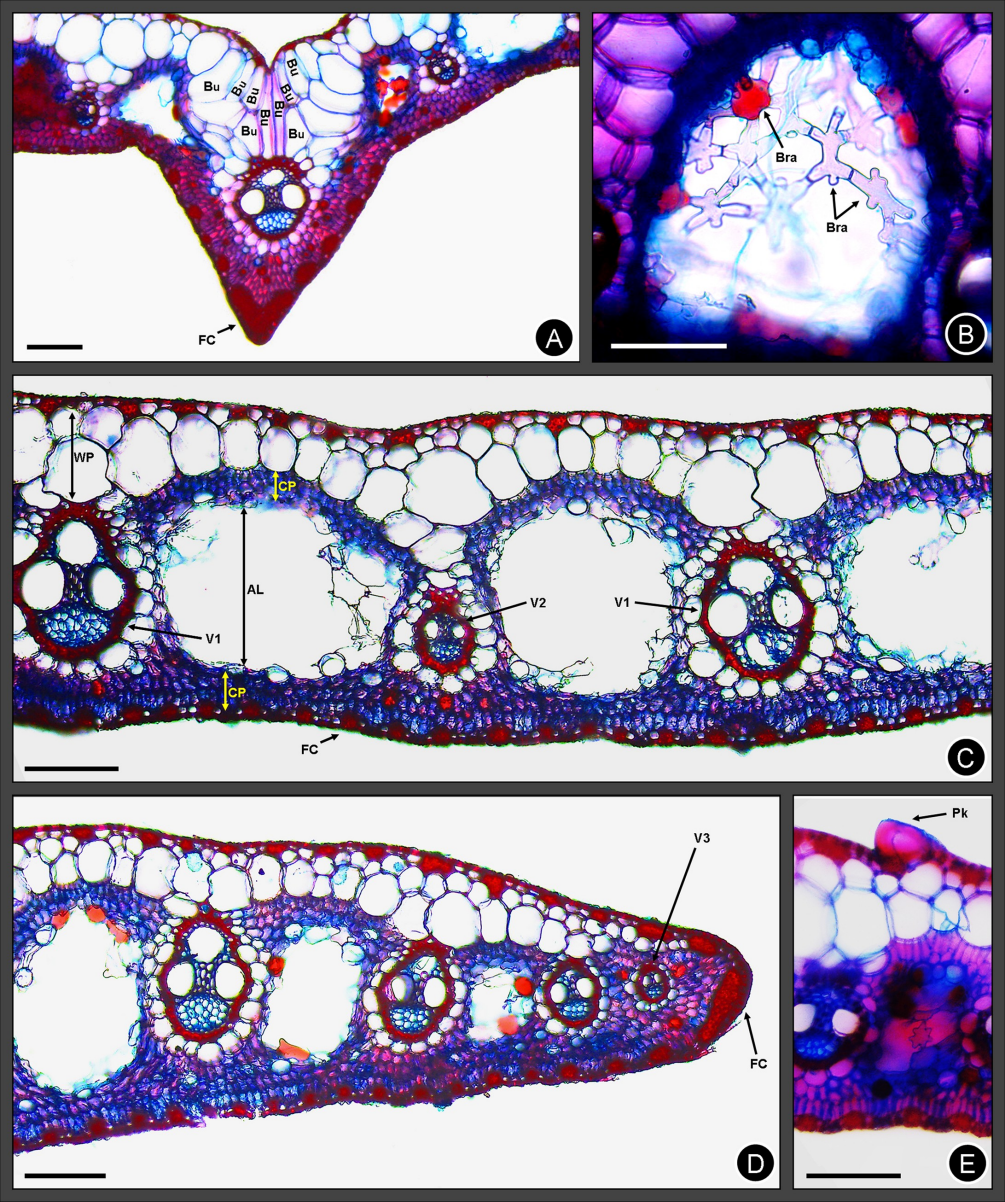
Light micrographs of transverse sections from the leaf blades of *Cyperus prophyllatus*, *sp*. *nov*. Taken from holotype *A*.*R*.*O*. *Ribeiro et al*. *487* (CVRD). (A) Midrib. (B) Detail of air lacuna showing braciform cells. (C) Arm of the leaf blade. (D) Leaf blade margin and its edge. (E) Detail of prickle double walled with thickened outer wall. AL = air lacuna, Bu = bulliform cell, Bra = braciform cell, CP = chlorophyll parenchyma, FC = fibre cap, Pk = prickle, V1 = 1^st^ order vascular bundle, V2 = 2^nd^ order vascular bundle, V3 = 3^rd^ order vascular bundle, WP = colorless parenchyma. Scale bars: A, C–E = 100 μm. B = 50 μm.

*Cyperus prophyllatus* A.R.O.Ribeiro, Pereira-Silva & M.Alves, sp. nov., [urn:lsid:ipni.org:names: 77216310–1] (Figs 1–7).

### Diagnosis

*Cyperus prophyllatus* is similar to *C*. *blepharoleptos* Steud. and *C*. *gardneri* Nees, from which it differs by hollow rhizome internodes; ligule present; spikelet prophyll conspicuous; rachilla articulate below spikelet prophyll with the spikelet falling attached to its prophyll at base; rachilla articulation 0.2–0.4 mm long, corky, tumid, protuberant, semiring to ring-shaped, yellowish to reddish, persistent at the base of the spikelet prophyll; glumes persistent; style 3-fid, persistent; achene trigonous, slightly compressed, surface approximately smooth, muticous or mucronate, mucron up to 0.1 mm long.

### Type

BRAZIL: Espírito Santo: Linhares, Reserva Natural Vale, Rio Barra Seca, próximo a foz, very frequent, 9 m, 19°05’03.4”S, 39°53’04.7”W, 25 September 2019, fl. and fr., *A*.*R*.*O*. *Ribeiro*, *V*.*S*. *Ribeiro & G*. *Felitto 487* (HOLOTYPE: CVRD!; ISOTYPES: B!, EAC!, FLOR!, ILL!, K!, MO!, MOSS!, MW!, NY!, P!, PE!, RB!, SAMES!, SP!, UB!, UFP!).

### Description

Perennial herb, floating aquatic, 115–140 cm tall. Rhizome leptomorph, floating, 5–12 mm wide, surface iridescent, rhizome internodes hollow, 4–85 mm long, rhizome nodes solid; cataphylls 22–76 × 13–19 mm, ovate to lanceolate, apex rounded to acuminate, muticous to aristate, vinaceous, mucron or arista up to 16 mm long. Culm 108–130 cm × 6–12 mm, trigonous, smooth to antrorsely scabrous on angles at apex, glabrous, transverse septa absent, hollow in the basal internodes, solid in the apical internodes and all nodes. Leaf blade 45–81 cm × 7–14 mm, linear-triangular, conduplicate, chartaceous, green, papillose with incomplete cross veins on abaxial surface when dried, smooth, rarely papillose on adaxial surface, antrorsely scabrous on margins and midrib on abaxial surface of the medial and the apical third, apex acuminate; ligule 0.5–1.2 mm long, charthaceous, ciliate at apex, reddish ferrugineous to maroon; leaf sheath 4.5–11.0 cm long, chartaceous, glabrous to rarely ciliate in the joint with leaf blade, sheath orifice in V-shaped to concave, glabrous; sheath margins membranaceous with a obtuse apex. Inflorescence bracts 8–12, 1.2–44 cm × 1.5–11.0 mm, linear to linear-triangular, indument similar to leaves; base of the bracts with auricules obtuse, membranaceous to hyaline. Inflorescence 7.0–13.5 × 5.5–12.0 cm; 3–4 orders of branches, anthelodium on first order, anthelodium, spike or subdigitate spike on second order and third order, spike or subdigitate spike on ultimate order. Primary rays 10–14, 0.5–7.0 cm long, subtended by a sheathing prophyll. Secondary bracts 4.5–13.0 × 1.5–2.0 mm. Secondary rays absent or present, up to 23 mm long, subtended by a sheathing prophyll with a tumid basal articulation, yellowish to reddish. Spikes 3.5–9.0 × 5.0–12.0 mm, hemispheroid to widely ovoid. Rachis 0.6–3.3 mm long, visible. Spikelet bract 1.2–2.3 × 0.7–1.2 mm, margins ciliate, apex acute to rounded, mucronate to aristate, mucron or arista 0.3–1.4 mm long. Spikelet prophyll 1.6–2.2 × 1.3–2.0 mm, conspicuous, bicarinate, apex rounded, obtuse, retuse to cordate, ciliate at margins. Spikelets 3–8 per spike, 4.0–7.5 × 1.4–2.8 mm, 0.9–1.0 mm thick, lanceoloid, laterally compressed, ratio wide: thick 1.5–2.8: 1. Rachilla articulate below spikelet prophyll with spikelet falling attached to its prophyll at base (very rarely a cluster of 2 spikelets falling attached to a single prophyll at base); rachilla internodes 0.4–0.6 mm long; rachilla wings 0.2–0.3 mm wide, chartaceous, pale orange to reddish; rachilla articulation 0.2–0.4 mm long, corky, tumid, protuberant, semiring to ring-shaped, yellowish to reddish, persistent at base of spikelet prophyll. Glumes 4–11, 1.8–3.0 × 1.6–2.4 mm, persistent, appressed, ovate, ciliate at margins, glabrous on the surface and carina, unicarinate, medially 9–15-nerved (including carina), apex obtuse to rounded, mucronate to aristate, margins reddish to maroon, carina greenish to ochraceous, mucron or arista 0.5–0.8 mm long, straight to slightly recurvate up to a 20° angle. Stamens 3; anther 1.5–2.0 × 0.2–0.3 mm, yellowish to ferruginous, connective prolongation present, 0.1–0.2 mm long, pale to whitish, glabrous to antrorsely scabrous. Style 1.6–1.9 mm long, persistent; stigmas 3, 0.7–1.3 mm long, base persistent. Achene 1.2–1.5 × 0.7–0.8 mm, trigonous, slightly compressed, 0.5–0.6 mm thick, obovoid to ovoid, apex acute and mucronulate, ochraceous to ivory, surface approximately smooth, mesocarp corky, completely covered by glume, mucron up to 0.1 mm long.

### Distribution

At present, *C*. *prophyllatus* is known only from the aquatic vegetation of the phytophysiognomy of Seasonal Semideciduous to Evergreen Forest, belonging to the Atlantic Forest in the Reserva Natural Vale, Espírito Santo State, Southeastern Brazil (Fig 3). The geographical distribution of related species *C*. *blepharoleptos* and *C*. *gardneri* are wider than *C*. *prophyllatus*. Whilst *C*. *blepharoleptos* can inhabit margins of waterbodies as well as be a floating aquatic macrophyte in tropical and subtropical areas of Africa and America, *C*. *gardneri* grows exclusively as a floating aquatic macrophyte in perennial rivers, lakes, or lagoons only in Neotropics (Fig 3).

### Conservation status

*C*. *prophyllatus* appears to have a restricted distribution, being known only from a few subpopulations from Reserva Natural Vale. This species is estimated to have an extent of occurrence (EOO) and area of occupancy (AOO) of 12 km^2^, and its geographic range is restricted to less than five locations. According to criteria proposed by IUCN [44], *C*. *prophyllatus* has ecological parameters that could belong to two categories: Endangered (EN) due to AOO (12 km^2^) between 10 km^2^ and 100 km^2^ or Critically Endangered (CR) due to EOO (12 km^2^) less than 100 km^2^. Nevertheless, the IUCN [44] recommends choosing from the higher risk category for a more precautionary approach to making urgent decisions based on limited information. Although *C*. *prophyllatus* occurs in a Protected Area (PA) with population greater than 400 individuals, the surroundings of Reserva Natural Vale are under pressure due to fragmentation caused by urban development and agriculture, like most of the range of the Atlantic Forest [63]. Therefore, *C*. *prophyllatus* can be preliminary considered Critically Endangered (CR) B1ab(iii), while more studies are required to expand the botanical collection effort and increase the knowledge about its geographic range.

### Phenology

Flowering and fruiting collections were made within July and October.

### Etymology

The name of the specific epithet refers to the conspicuous spikelet prophyll that remains attached to the base of the spikelet after disarticulation of the rachilla (Figs 1E, 1F, 2I, 2J and 5E). Moreover, *C*. *prophyllatus* has the rachilla articulation with 0.2–0.4 mm long, corky, tumid, protuberant, semiring to ring-shaped, yellowish to reddish, persistent at the base of the spikelet prophyll (Figs 1E, 1F, 2I, 2J and 5E), which are exceptional characteristics that become *C*. *prophyllatus* considerably distinct from all other known species of *Cyperus*.

### Morphologically related species

*Cyperus prophyllatus* is morphologically similar to *C*. *blepharoleptos* and *C*. *gardneri* from which it differs by the morphology of its rhizome, ligule, leaf blade, inflorescence type, spikelet disarticulation pattern, rachilla articulation, glumes, style, and achene (Table 2). In *C*. *prophyllatus*, the rhizome (Figs 1A, 2E and 2F) has hollow internodes (spongy in *C*. *blepharoleptos* and *C*. *gardneri*), the ligule (Figs 1B, 2G and 5D) is present (absent in *C*. *gardneri*), the inflorescence (Figs 1C, 1D and 2H) has 3–8 spikelets per spike on ultimate order branches (15–70 in *C*. *blepharoleptos*, 10–32 in *C*. *gardneri*), the spikelet prophyll (Figs 1E, 1F, 2I, 2J and 5E) is conspicuous and remains attached to the base of the spikelet after rachilla disarticulation at maturity (absent in *C*. *blepharoleptos* and inconspicuous or not persistent at the base of the spikelet in *C*. *gardneri*), the rachilla articulation (Figs 1E, 1F, 2I, 2J and 5E) is corky, tumid, protuberant, semiring to ring-shaped, yellowish to reddish, and persistent at the base of the spikelet prophyll after the disarticulation (rachilla articulation is absent in *C*. *blepharoleptos* and absent or when present is flat, not protuberant, and not persistent at the base of the spikelet in *C*. *gardneri*), the anther (Fig 2H) has 1.5–2.0 mm long (0.3–0.7 mm long in *C*. *gardneri*), the style (Fig 1G) is 3-fid, persistent (2-fid, deciduous in *C*. *blepharoleptos* and 3-fid, deciduous in *C*. *gardneri*), the achene (Figs 1G, 2K and 6A–6D) is trigonous, surface approximately smooth, mucron up to 0.1 mm long (lenticular, mucron or arista 0.2–0.7 mm long in *C*. *blepharoleptos* and surface approximately smooth at apex and with one central puncticulate depression area in each side at base in *C*. *gardneri*).

**Table 2:** Comparison of *Cyperus prophyllatus* with allied species in *C*. sect. *Oxycaryum*.

	*C*. *prophyllatus sp*. *nov*.	*C*. *blepharoleptos*	*C*. *gardneri*
**Rhizome internodes**	Hollow	Spongy	Spongy
**Leaf blade**	Chartaceous, 7–14 mm wide	Membranaceous to chartaceous, 2.8–10 mm wide	Membranaceous, 2–3.8 mm wide
**Leaf ligule**	Present	Present	Absent
**Inflorescence**	Spike or subdigitate spike on ultimate order, 3–8 spikelets per spike	Capitate spike on ultimate order, 15–70 spikelets per spike	Capitate spike on ultimate order, 10–32 spikelets per spike
**Spikelet prophyll**	Conspicuous, 1.3–2 mm wide	Absent	Inconspicuous, 1–1.2 mm wide
**Spikelet disarticulation pattern**	Spikelet falling as a unity attached to its prophyll at base (rarely a cluster of 2 spikelets falling attached to a single prophyll) with a corky rachilla articulation persistent at the base of the spikelet prophyll	Spikelet and glumes persistent, achenes deciduous, entire inflorescence disarticulating belatedly after the fall of the achenes	Glumes gradually deciduous from the base to the apex of the spikelet with the rachilla disarticulating belatedly after the fall of the glumes
**Rachilla**	Rachilla articulation 0.2–0.4 mm long, corky, tumid, protuberant; rachilla internodes 0.4–0.6 mm long	Rachilla articulation absent, rachilla internodes 0.2–0.3 mm long	Rachilla articulation absent, rarely present, up to 0.1 mm long, flat; rachilla internodes 0.3–0.4 mm long
**Glume**	Glumes distichous arranged	Glumes spirally arranged	Glumes distichous arranged
**Anther**	1.5–2.0 mm long	(0.6–)1–2.1 mm long	0.3–0.7 mm long
**Style**	3-fid, 1.6–1.9 mm long, persistent	2-fid, 0.9–1.5 mm long, deciduous	3-fid, 0.9–1.3 mm long, deciduous
**Achene**	1.2–1.5 × 0.7–0.8 mm, trigonous, slightly compressed, 0.5–0.6 mm thick, mucron up to 0.1 mm long, surface approximately smooth or with 1–5 grooves at base	1.5–2.4 × 0.7–0.9 mm, lenticular, 0,2–0,5 mm thick, mucron or arista 0,2–0.7 mm long, surface approximately smooth at base	1.3–1.5 × 0.7–1 mm, trigonous, not compressed, 0,7–1,0 mm thick, mucron up to 0.1 mm long, surface approximately smooth with a puncticulate depression area in each side at base

### Additional collections (paratypes)

BRAZIL: Espírito Santo State: Linhares, Reserva Natural Vale, estrada Jueirana Vermelha, Rio Barra Seca, 19°05’01.6”S, 39°53’03,7”W, 26 August 2019, fl., *G*.*S*.*Siqueira & G*. *Felitto 1317* (CVRD!); próximo a foz do Rio João Pedro, perto da casa no final da Estrada Farinha Seca, 19°11’09.3”S, 39°54’19.8”W, 24 September 2019, *A*. *R*. *O*. *Ribeiro*, *V*.*S*. *Ribeiro & G*. *Felitto 490* (EAC!); Reserva Natural Vale, Estrada Farinha Seca, final da estrada. Km 4.6, 14 October 1998, fl. and fr., *D*.*A*. *Folli 3253* (CVRD!, MOSS!, UFP!); Reserva Natural Vale, Final da Estrada da Bomba D’água, 11 July 2003, fl., *D*.*A*. *Folli 4544* (CVRD!, MOSS!, UFP!).

### Phylogenetic relationships

Phylogenetic trees resulting from ML and BI analyses of the concatenated dataset recovered congruent topologies. In general, relationships in C_3_ *Cyperus* Grade are strongly supported, whereas relationships are poorly supported in the C_4_ *Cyperus* Clade (Fig 4). *Cyperus prophyllatus* is resolved in a clade with *C*. *blepharoleptos* and *C*. *gardneri* that represents *Cyperus* sect. *Oxycaryum* (Nees) Larridon of the C_3_ *Cyperus* Grade (Fig 4).

### Identification key to the species of *Cyperus* sect. *Oxycaryum* (Nees) Larridon

1. Rhizome internodes hollow; spikelet prophyll conspicuous, 1.3–2.0 mm wide; spikelet falling as a unity attached to its prophyll at base (rarely a cluster of 2 spikelets falling attached to a single prophyll); rachilla articulation 0.2–0.4 mm long, corky, tumid, protuberant, persistent at the base of the spikelet prophyll; style persistent on the achene apex *C*. *prophyllatus*

1’. Rhizome internodes spongy; spikelet prophyll absent or inconspicuous, 1.0–1.2 mm wide; spikelet and glumes persistent, achenes deciduous, entire inflorescence disarticulating belatedly after the fall of the achenes or glumes gradually deciduous from the base to the apex of the spikelet with the rachilla disarticulating belatedly after the fall of the glumes, without spikelet prophyll attached at base; rachilla articulation absent, rarely present, up to 0.1 mm long, flat, not persistent at the base of the spikelet prophyll; style deciduous 2

2. Leaf ligule absent; glumes distichously arranged; style-branches 3; achene trigonous..........................................

................................................................................................................................*C. gardneri*

2’. Leaf ligule present; glumes spirally arranged, style-branches 2, achene lenticular

................................................................................................................................*C. blepharoleptos*

### Micromorphology observed in Scanning Electron Microscopy (SEM)

In the abaxial surface of the leaf blade, the intercostal zone has one or two rows of diacytic stomata intercalated with common epidermal cells with papillose wall. The stomata are present only in the abaxial side (Fig 5B), which classify the leaf as hypoestomatic. The costal zone contains two rows of thick-walled epidermal cells, tabular or square-shaped with one or two papillae with base entire or stellate per cell (Fig 5B). The leaf scabrosity is due to the antrorse prickles which are present in leaf margins and in the costal zone of the midrib (Fig 5C).

In the adaxial surface of the leaf blade (Fig 5A), the intercostal zone has common epidermal cells with papillose wall (Fig 5A). The costal zone possesses two rows of thick-walled epidermal cells tabular or square-shaped with one or two papillae with base entire or stellate per cell interrupted by common epidermal cells smooth-walled (Fig 5A). Antrorse prickles are present in costal and intercostal zones (Fig 5A). Ligule has ciliate single-celled hairs abundant on the apex (Fig 5D).

In the spikelet prophyll (Fig 5E), the costal zone contains two rows of thick-walled epidermal cells tabular or square-shaped with one or two papillae with base entire or stellate per cell (Fig 5E). The rows of epidermal cells tabular or square-shaped are in similar position to the ribs (nerves) of the spikelet prophyll (Figs 2I, 2J and 5E). Stomata are scarce and present only next to the two carinas of the spikelet prophyll (Fig 5E). Common epidermal cells have abundant papillae on the cell wall (Fig 5E), which are similar in morphology to the leaf blade. The margins of the spikelet prophyll have abundant ciliate single-celled hairs, mainly on the apex (Fig 5E).

In the glume (Fig 5E–5H), the coastal zone has two or three rows of thick-walled epidermal cells tabular or square-shaped with one or two papillae with base entire or stellate per cell (Fig 5G). The rows of epidermal cells tabular or square-shaped are in similar position to the ribs (nerves) of the glume (Figs 2I, 2J and 5G). Stomata are abundant next to the regions of the carina and mucron or arista, but they are absent or scarce in other regions of the glume (Fig 5F and 5H). Common epidermal cells have abundant papillae on the cell wall (Fig 5G), which are similar in distribution and morphology to the leaf blade and spikelet prophyll. The margins of the glume are ciliate with single-celled hairs, except in the region of the mucron or arista, which is glabrous (Fig 5E–5H).

Achenes (Fig 6A–6D) are approximately smooth with slight sinuosities on anticlinal walls on the middle third and most of the surface of the basal and apical third (Fig 6A–6D). The sinuosities at base (Fig 6A and 6C), is more prominent on the anticlinal walls and 1–5 grooves occur with reticulate depressions and protuberant anticlinal walls. The apex (Fig 6A and 6D) also contains sinuosities more prominent next to the style insertion and reticulate depressions have protuberant anticlinal walls. Papillae, hairs and silicified cells are absent on achene surface.

### Anatomy

In the transverse section, the radially arranged and elongated mesophyll cells of the chlorophyll parenchyma are absent around the vascular bundles (Fig 7A, 7C and 7D) in all accessions analyzed as well as the Kranz sheath (Fig 7A, 7C and 7D). The colorless parenchyma surrounds the vascular bundles and has thin-walled cells that are larger in the region close to the adaxial side (Fig 7A, 7C and 7D). In lacunosous parenchyma, several braciform cells (Fig 7B) were observed in large air lacunae surrounded by chlorophyll parenchyma (Fig 7A–7D).

The cuticle (Fig 7A and 7C–7E) is thin in the both abaxial and adaxial sides and leaf blade is V-shaped in cross section (Fig 7A). Epidermis is unistratified with common epidermal cells round shaped and greater in the adaxial side (Fig 7A and 7C–7E). The leaf scabrosity is due to the prickles (Figs 5A, 5C and 7E), which are often distributed next to the midrib, bulliform cells, and on the edge of the leaf margin (Figs 5A, 5C and 7E). Prickles are acute, have enlarged base and thickened outer wall (Figs 5A, 5C and 7E). Bulliform cells fill up to half of the mesophyll (Fig 7A) and occur only in the adaxial side of vascular bundle on the midrib. Fibre caps (Fig 7A, 7C and 7D) have strongly thickened wall and occur in the mesophyll next to adaxial and abaxial epidermis along the entire leaf surface.

The vascular system (Fig 7A, 7C and 7D) comprises collateral vascular bundles from 1^st^ to 3^rd^ order elliptical, circular or oval. The vascular bundle sheath (Fig 7A, 7C and 7D) is doubled in all orders, the inner sheath is complete and formed by sclerenchyma, and outer one is often incomplete, sometimes complete and always constituted by parenchyma.

The midrib (Fig 7A) has a V-shaped and a prominent keel and a colorless parenchyma more developed in the adaxial side (Fig 7A). The vascular system from the midrib comprises one of 1^st^ order vascular bundle and two of 2^nd^ order (Fig 7A). The 1st order one is rounded, central, and next to the abaxial side (Fig 7A).

The leaf blade margins can be from acute to rounded (Figs 5C and 7D). The vascular system of the leaf blade margins is composed by one of 2^nd^ order and one of 3^rd^ order (Fig 7D). The edge of the leaf margin (Fig 7D) contains fibres forming a thick cell caps and scabrosity constituted by prickles or hooks, both with base not bulbous (Figs 5C and 7D).

## Discussion

The new species *Cyperus prophyllatus* is unique among the c. 950 species of the genus *Cyperus*, being recognized by a exceptional spikelet disarticulation pattern that includes a combination of the following characters: spikelets 3–many-glumed; a conspicuous spikelet prophyll that remains attached to the base of the spikelet after rachilla disarticulation at maturity; rachilla articulation 0.2–0.4 mm long, corky, tumid, protuberant, semiring to ring-shaped (Figs 1E, 1F, 2I, 2J and 5E), yellowish to reddish (2I-J), and persistent at the base of the spikelet prophyll after the disarticulation [10–13,15,16,19,21]. *Cyperus* sect. *Neohemicarpha* Bauters and *C*. sect. *Lipocarpha* (R.Br.) Bauters also hold species with the spikelet deciduous as a single unity attached to its prophyll at base, but in those sections the spikelet prophyll is inconspicuous and spikelets are reduced to a single glume (sometimes absent) covered by spikelet bract [9,13].

According to our molecular phylogenetic results (Fig 4), *C*. *prophyllatus* emerges in the C_3_ *Cyperus* Grade, which is morphologically heterogeneous and characterized by C_3_ photosynthetic pathway [3,8,10,11]. Within the C_3_ *Cyperus* Grade, our phylogenetic study supports the placement of *C*. *prophyllatus* in the clade representing *Cyperus* sect. *Oxycaryum*, that also holds *C*. *blepharoleptos* and *C*. *gardneri* (Fig 4). Morphological characters of *C*. *prophyllatus* such as an aquatic floating habit, 3 stamens, and a corky achene are shared with the other species of the section [10,11]. After the discovery and inclusion of *C*. *prophyllatus*, the synapomorphies of the section *Oxycaryum* are the presence of ciliate single-celled hairs in both spikelet prophyll and glumes (Figs 1E, 1F, 2I, 2J and 5E) and corky achenes (1G, 2K, 6A–D) [10,11]. Furthermore, mucronate or aristate glumes with a mucron or arista longer than 0.3 mm (Figs 1F, 2I and 5F) is also shared by species of section *Oxycaryum*, although it should not be considered as apomorphy to this clade [10,11]. The charthaceous leaf ligule with a ciliate apex is very unusual in *Cyperus* [10,15,16,19–25], being reported only in *C*. *prophyllatus* and *C*. *blepharoleptos*.

Anatomical data also corroborates the placement of *C*. *prophyllatus* in C_3_ *Cyperus* Grade. The absence of the radial chlorophyll parenchyma and Kranz sheath are associated to the eucyperoid anatomy type as well corresponds to the C_3_ photosynthetic pathway [3,8,10,11,16,64,65]. The new species shows other anatomical characteristics observed in C_3_ *Cyperus* Grade as a colorless parenchyma surrounding the vascular bundles and large air lacunae immerse in chlorophyll parenchyma in mesophyll tissue [64,66,67]. The air lacunae make survival in aquatic environments possible, since it promotes oxygen flow from leaves to submerged organs [68–70]. In allied species *C*. *blepahroleptos*, braciform cells also were observed in air lacunae [67]. The braciform cells provide structural support to air lacunae as well partake in the gas flow among distinct tissues of aquatic plants [67,71–76].

Other structures present in *C*. *prophyllatus* are recurrent linked to efficiency of sedges from aquatic environments such as achene and rachilla articulation corky, and hollow rhizome. Corky or spongy thickenings in achenes, rachilla internodes or glumes enable the diaspore floatation in species of section *Oxycaryum* and have been reported in species of other *Cyperus* sections as *C*. *pectinatus* Vahl and *C*. *pedunculatus* (R.Br.) J.Kern [10,16]. Hollow internodes in the rhizome have not been reported yet in *Cyperus*, although hollow culms already have been observed in other genera of Cyperaceae [16,19]. The hollow rhizome keeps large air cavities even when submerged and would allow aquatic species to float in its native environments [77–79].

The micromorphology of *C*. *prophyllatus* observed by SEM revealed the presence of hypoestomatic leaves and antrorse prickles in the leaf blade margins, which has already been reported in other *Cyperus* species [64,67,80–82]. Denton [66] recorded to Luzulae Group (informal group), C_3_ *Cyperus* Grade, similar features found in the new species as two rows of thick-walled epidermal cells tabular or square-shaped with one or two papillae with base entire or stellate per cell as well as common epidermal cells with papillose wall. In *C*. sect. *Oxycaryum*, similar characteristics as the presence of ciliate single-celled hairs in both spikelet prophyll and glumes, as well achene approximatelly smooth on surface of the middle third had already been observed also in *C*. *blepharoleptos* and *C*. *gardneri* [10]. Although both *C*. *prophyllatus* and *C*. *gardneri* have achene trigonous, it is possible to differentiate them by the achene base which in *C*. *prophyllatus* is approximately smooth or with 1–5 grooves, as in *C*. *gardneri* there is a puncticulate depression area in each side at base [10].

Our ecological data show that *C*. *prophyllatus* is an endangered and endemic species from the Atlantic Forest in Southeastern Brazil. It occurs in the Reserva Natural Vale, a private Protected Area (PA) in Espírito Santo State with a high number of endemic plant and animal species [83–86]. In most PAs in Brazil, the knowledge about the flora is still incomplete or nonexistent [87,88]. Nevertheless, PAs can be threatened in Brazil by reduction or even extinction of species not yet discovered due to the corporate and political lobbying and loss of vegetation cover by anthropogenic interference [36]. Whereas several Protected Areas are threatened in Brazil, botanical and taxonomic studies using integrative approach combining analyses of multiple data sources are fundamental to reinforce and help the continuity and effectiveness of biodiversity conservation.
